# Assessment of intimate partner violence victimization and its association with the psychological state of abused women and social support in Saudi Arabia: a cross-sectional study

**DOI:** 10.1186/s12889-024-20698-0

**Published:** 2024-12-20

**Authors:** Adnan Innab, Wejdan Shaqiqi, Kamila Alammar, Alkadi Alshammari, Rawan Shaqiqi

**Affiliations:** 1 Nursing Administration and Education Department, College of Nursing, King Saud University, Riyadh, 12372 Saudi Arabia; 2 College of Nursing, King Saud Bin Abdulaziz University for Health Sciences, Riyadh, Saudi Arabia; 3 King Abdullah International Medical Research Center, Riyadh, Saudi Arabia; 4 SURGCA. Ambulatory Surgical Center, 13317 Riyadh, Saudi Arabia; 5 Community, Psychiatric and Mental Health Nursing, King Saud University, Riyadh, Saudi Arabia; 6 Emergency Department, Ministry of Health, Jeddah, Saudi Arabia

**Keywords:** Intimate partner violence, Psychological distress, Anxiety, Depression, Social support

## Abstract

**Background:**

Intimate partner violence (IPV) against women is a significant global concern, profoundly affecting physical, psychological, sexual, and financial well-being. Its prevalence is notably high in conservative societies including Saudi Arabia (SA). Given the limited research on the role of social support in IPV within SA. This study aimed to assess IPV victimization and its association with the psychological state of abused women in SA, and the effects of social support on the women’s experience of IPV.

**Methods:**

A total of 128 women participated in this cross-sectional study. Data were collected from September 2022 to March 2023 using the World Health Organization Multi-Country Violence Against Women, the General Health Questionnaire, and the Multi-Dimensional Scale of Perceived Social Support.

**Results:**

One-quarter of women experienced at least one type of IPV, with more than half of them experienced financial (65.6%), sexual (53.9%), emotional (57.8%), controlling behavior (43.8%) and physical (39.8%) abuse. IPV victimization, be it physical, emotional, or sexual abuse, and IPV frequency were positively correlated with psychological distress (M = 15.05, SD = 6.82, *p* = .009), anxiety and depression (M = 6.29, SD = 2.57, *p* = .004), and loss of confidence (M = 2.57, SD = 1.90, *p* < .001). Social support was negatively associated with physical and controlling behavior abuse (*p* = .01) as well as IPV frequency (*p* = .024). The risk factors of IPV were unemployed women, employed husbands, history of child abuse for wives and husbands, financial struggle and arranged marriage.

**Conclusions:**

The conclusion was that IPV can cause psychological distress, anxiety, depression, and loss of confidence and can be buffered by social support. Given its prevalence and impact on mental health, it is crucial to establish strict policies and regulations to prevent IPV and provide effective interventions and support for abused women.

## Background

Intimate partner violence (IPV) is defined as any physical, sexual, psychological, or economic act intended to cause harm or exert control over an intimate partner [1]. It is a global public problem that threatens women physically, psychologically, sexually, and financially. Worldwide, approximately one-third of women experience IPV, with variations in prevalence and form across different countries [1]. Studies indicated that the prevalence can be higher in conservative societies. For instance, in Ethiopia, 51.8% of partnered women reported experiencing IPV in past 12 months [2]. Similarly, a systematic review revealed a pooled prevalence of 31% during COVID-19 pandemic, with the highest rates in developing regions (33%), particularly in Uganda (68%), and the lowest in the USA (10%) [3] A secondary analysis of population based surveys (2000 to 2021) across 53 low- and middle- income countries reported a weighted prevalence of 37.2% among women aged 15 to 49 years, with an increasing trend observed in six countries, ranging from a 1.2% rise in Nigeria to a 6.6% rise in Sierra Leone (Ma et al., 2023) [4]. Nevertheless, IPV prevalence is likely to be underestimated because it is often underreported.

In the Middle East region, the overall prevalence of IPV has been reported at 26.3%, with psychological abuse being the most common form (48.6%). Other types of abuse include physical (28.4%), economic (19%), and sexual (18.4%) [5]. In Saudi Arabia, a recent study found that approximately 14% of 2254 women had experienced IPV [6], a lower figure than that reported in an earlier systematic review, indicating 39% to 45% of women were abused by their husbands in Saudi Arabia [7]. This variation could be attributed to the increasing efforts of the government to empower women and strengthen their rights in all sectors, such as education, justice, business, and politics. For example, the ratio of employed women has increased from 27.6% to 30.4% and women’s participation in the labor force has reached 36% of the total working population [8]. This could enhance women's financial autonomy and participation in decision-making. The Saudi government established the National Family Safety Program in 2005 to stop domestic violence by raising awareness at the individual and community levels about its harmful effects and negative consequences [9]. Cases are also reported to the government through a unified national notification number and victims are provided with the appropriate social and health care in addition to protection and shelter. Furthermore, support systems and care programs have been developed to ensure adequate physical, emotional, and financial support and care for women, especially vulnerable groups such as widows and abused women [10].

However, despite such government efforts, IPV remains prevalent, and the stigma associated with IPV probably means that it is underreported. Some researchers report that IPV is seen as a family issue that should be dealt with privately, while other researchers highlight the fact that Saudi society is a collective community in which group needs are prioritized over those of the individual [11, 12]. Wali et al. (2020) indicated that Saudi women are expected to obey their husbands and protect their family unit even when enduring abuse, while other researchers reported that social stigma still surrounds divorce and chastity [13]. Women’s families may not support divorce, may refuse to take their divorced daughters in, and may even blame their daughter for the abuse they suffered [14]. The authors added that a woman living alone is considered socially unacceptable and is an economic burden on women [14]. These issues, along with conflicting feelings of guilt and loyalty, affect women’s ability to report abuse or leave their husbands, especially in conservative communities.

Scholars have stated that IPV is associated with long-term mental health problems, including anxiety and depression [15]. Other researchers conducted a literature review and concluded that victim-blaming and negative reactions resulted in increased psychological health symptoms and a poorer quality of life [16]. Although Fortin et al. (2012) and Ogbe et al. (2020) indicated that social support can ameliorate such impacts, the latter review found that sociocultural factors and victim perceptions of IPV prevented many women from seeking help [17, 18]. Moreover, abusers’ continued control over their victims further inhibits help-seeking behaviors and resettlement.

Previous Saudi-based studies have largely focused on the prevalence of IPV and its impact on victims’ health; therefore, this study fills a gap by investigating the functionality of social support for IPV victimization.

### Purpose statement

Given the dearth of evidence regarding the impact of social support on IPV in Saudi Arabia, it is imperative to further investigate this issue within the specific cultural and social context. Addressing this research gap is critical for understanding how social support may influence the prevalence, experiences, and psychological outcomes of IPV in Saudi Arabia, as well as for developing targeted interventions and policies that meet the needs of the population. The substantial burden of IPV underscored the importance of this study, especially in the light of the Sustainable Development Goal 5.2, which emphasizes the elimination of all forms of violence against women. Achieving this goal requires thorough examination of factors that affect and moderate IPV. Therefore, this study aimed to assess the prevalence of various forms of IPV, including experience of physical, emotional, sexual, financial and controlling behaviors and its impact on the psychological state of women living in Saudi Arabia. It also investigated the role of social support on abused women’s experience of IPV and their psychological health. The specific objectives to: 1) to compare the psychological distress, anxiety and depression, social dysfunction, loss of confidence, and perceived social support between women those who had experienced any type of IPV and those who had not; 2) to determine the correlation between anxiety and depression, social dysfunction, loss of confidence, and support from family, friends, and husband; and 3) to evaluate the IPV differences in IPV victimization, psychological distress and perceived social support based on participants’ demographic characteristics.

## Methods

### Study design

A cross-sectional, correlational, descriptive design was used. This study adhered to the Strengthening the Reporting of Observational Studies in Epidemiology (STROBE) framework. An online survey was distributed to the participants through social media to enhance recruitment from different regions of Saudi Arabia.

### Study participants

Social media platforms (Twitter, Facebook, and WhatsApp) were used to recruit participants from different regions of Saudi Arabia. The inclusion criteria were as follows: women living in Saudi Arabia who were at least 18 years old, married or previously married, and who had signed an electronic consent form by ticking a box. Those who were single and had never been married or were not living in Saudi Arabia were excluded from the study. Participants were recruited using a snowball sampling method. The sample size was calculated using G*Power software 3.1. For a significance level of 0.05, power of 0.8, an effect size of 0.15, and seven predictors, a minimum sample size of 92 was required to run the inferential statistics. In addition to the determined minimum sample size, we aimed to recruit an additional 15% of participants to mitigate any issues associated with missing data. A total of 128 participants completed the questionnaires.

### Data collection procedure

Saudi Arabia comprises 13 regions and 66 cities. The data were not limited to any particular area as the aim of this study was to explore IPV across the country. The recruitment statement was provided in the same online link as the survey to ensure that the participants understood the purpose of the study and the risks and benefits of participating. Those who met the inclusion criteria and agreed to participate were given full access to the survey. Data were collected between September 2022 and March 2023, using an online survey posted on social media. Furthermore, to enhance recruitment, participants were encouraged to inform others they knew who might have experienced IPV about the study. The survey was conducted in Arabic as this was the participants’ native language. The survey took participants 15 − 25 min to complete. The majority of participants were from the central (32.8%) or western region (31.3), followed by those from the eastern region (25%), while participants from the northern and southern regions represented the minority.

### Instruments

The survey consisted of four parts: demographic characteristics, and three validated scales (the World Health Organization (WHO) Multi-Country Violence Against Women, the General Health Questionnaire (GHQ), and the Multi-Dimensional Scale of Perceived Social Support (MSPSS). The demographic form was adapted from the WHO Multi-Country Violence Against Women. It included items on age, marital status, level of education, region, employment status, monthly income, duration of marriage, childhood experience of violence, and history of witnessing domestic violence.

Following the completion of the demographic form, participants completed the Arabic version of the WHO Multi-Country Violence Against Women [19]. The Arabic version of the questionnaire has been validated and used in a wide range of Arabic countries, including Saudi Arabia [7]. The questionnaire included five types of violence, namely: physical, emotional, sexual, financial, and controlling behavior violence. For each type of violence, participants were asked about their past or present experiences with IPV, frequency of IPV, severity, effect of the violence, and reasons for either asking or not asking for help. The frequency of each type of violence was measured as "always," "sometimes," "rarely," or "never." Responding to any of the statements with any answer except for "never" was considered experience of IPV.

Following the WHO Multi-Country Violence Against Women, the participants completed the GHQ-12, which measures psychological distress [20]. This tool was originally developed by Goldberg (1988) as a self-report, unidimensional model to measure psychological health [20]. It consists of three latent factors representing this model: social dysfunction, anxiety/depression, and loss of confidence [20]. The instrument is measured on a 4-point Likert scale (ranging from 0 to 3). The total scores range from 0 to 36, with higher scores indicating worse conditions [21]. This tool has demonstrated good psychometric properties and is a useful screening tool for assessing psychological distress and minor psychiatric morbidities [22]. The reliability and validity of the Chinese and English versions of the scale have been established [23, 24]. The GHQ-12 has been translated into Arabic [25], but to validate it for use among a sample of adult women in the Saudi context, the tool was retranslated and pretested in our study. Two researchers and bilingual experts translated and back-translated the questionnaires from English into Arabic. Face and content validity tests were performed to ensure that the items expressed the same meaning, and the final version was pretested after collecting data from 20 participants.

Lastly, the participants completed the Arabic version of the MSPSS developed by Zimet et al.(1988) measures perceived support provided by friends, family, and significant others [26, 27]. The tool consists of 12 items measured on a 3-point Likert scale (agree, neutral, or disagree) Higher scores indicate greater perceived social support. The Cronbach’s alpha of the Arabic version of the scale was 0.87 [27].

### Ethical considerations

Institutional Review Board approval was obtained from [deleted for peer review] prior to data collection. This study adhered to the principles of the Declaration of Helsinki. Permission to use the tools was obtained from all authors. The participants’ anonymity, confidentiality, risks, and benefits were clearly addressed in the recruitment statements. Participants were informed that their participation was voluntary and that they could stop filling out the questionnaire if they decided to withdraw from the study. The participants were also asked to stop completing the questionnaire if they experienced psychological or emotional discomfort. Participants were notified that their participation would be anonymous, and no identifying information would be collected. Signing the consent form was required before accessing the questionnaire. The recruitment statement included the contact number for the Domestic Violence Center in case women required assistance.

### Data analysis plan

Data were analyzed using IBM SPSS Statistics (version 28). Data management and cleaning were performed before conducting descriptive and inferential statistical analysis. Descriptive statistics (e.g., mean, standard deviation, and percentage values) were used to display demographic characteristics and item analysis. Internal consistency reliability was tested across all scales and subscales. Pearson’s correlation coefficient was used to determine the association between IPV risk factors, psychological distress, and perceived social support. An independent sample t-test and analysis of variance (ANOVA) were used to measure the relationship between demographic characteristics, risk factors for IPV, psychological distress, and perceived social support.

## Results

Table 1 presents the socio-demographic data of the 128 women in the sample and their experience of IPV. The participants’ ages ranged from 18 to 59 years, the largest age group being 30 − 39 years old (39.8%). Saudi participants represented 93.8% of the sample, with the majority living in either the central region (32.8%) or the western region (31.3%). Nearly 60% of the participants had an undergraduate degree and 113 (88.3%) were married. Participants who had a source of income represented 53.1% of the sample, with almost half earning a monthly income around 2800$ (46.1%), and 59.4% agreed that their monthly income was sufficient for them. The average marriage period for the participants was 14.89 (SD = 9.4) years, and 84.4% (*n* = 108) of the participants were still living with their husbands. Notably, 37.5% (*n* = 48) of participants had a history of child abuse and witnessing domestic violence (53.1%, *n* = 68).

**Table 1:** Socio-demographic Data of Participants and Their Experience of IPV (*N* = 128)

Variable (Range)	n (%) or M (± SD)
Age: 18–29	22 (17.2)
30–39	51 (39.8)
40–49	41 (32.0)
50–59	14 (10.9)
Nationality	
Saudi	120 (93.8)
Non-Saudi	8 (6.3)
Region	
Central region	42 (32.8)
Western region	40 (31.3)
Eastern region	32 (25.0)
Southern region	10 (7.8)
Northern region	4 (3.1)
Level of education	
High school	30 (23.4)
Undergraduate education	77 (60.2)
Higher education	21 (16.4)
Marital Status	
Married	113 (88.3)
Divorced	12 (9.4)
Working or having a source of income	68 (53.1)
Family's monthly income	
Less than 1400$	17 (13.3)
1400$ to 2800$	52 (40.6)
More than 2800$	59 (46.1)
Income is sufficient	
Yes	76 (59.4)
No	52 (40.6)
Marriage period	14.89 (9.4)
Chose husband	77 (60.2)
Living with husband	108 (84.4)
History of child abuse/Witnessing domestic violence	68 (53.1)
Husbaned's history of child abuse/Witnessing	57 (44,5)
**Experiences of IPV**	
None	12 (9.4)
One type	32 (25.0)
Two types	15 (11.7)
Three types	25 (19.5)
Four types	23 (18.0)
All types	21 (16.4)
**Experience with IPV types**	
Physical abuse	51 (39.8)
Controlling behavior	56 (43.8)
Sexual abuse	69 (53.9)
Emotional abuse	74 (57.8)
Financial abuse	84 (65.6)
**Physical abuse**	
Been injured as a result of beating	
Yes	12 (23.5)
No	31 (60.7)
Think beating affects physical or psychological health	
No	2 (3.9)
A little	4 (7.8)
A lot	42 (82.3)
Think beating affects your work	
No	6 (11.7)
Loss of ability to concentrate	14 (27.4)
No possibility of work/sick leave	4 (7.8)
Loss of confidence in my capabilities	14 (27.4)
Spoke about the physical abuse to	
No one	15 (29.4)
Family	17 (33.3)
Friends	7 (13.7)
Relatives	3 (5.8)
Police	1 (1.9)
Anyone tried to help	
Family	15 (29.4)
Friends	5 (9.8)
Relatives	6 (11.7)
Reason for asking for help	28 (54.9)
Couldn't endure more	6 (21.4)
My children are suffering	6 (21.4)
Encouragement from friends	2 (7.1)
The injuries were severe	2 (7.1)
He threatened to kill me	2 (7.1)
He beat the children	1 (3.5)
Didn't ask for help	15 (29.4)
I am afraid of losing the children	12 (80)
Afraid of negative reputation for family	5 (33.3)
Other women did not receive support/help	2 (13.3)
Discussed together	2 (13.3)
Afraid of threats/more violence	2 (13.3)
I am afraid of divorce	1 (6.6)
It is normal/nothing serious	1 (6.6)

Financial abuse was the most prevalent type of IPV (65.6%, *n* = 84), indicating a significantly high prevalence of this particular form of abuse, followed by emotional abuse (57.8%, *n* = 74), sexual abuse (53.9%, *n* = 69), controlling behavior (43.8%, *n* = 56), and physical abuse (39.8%, *n* = 51), or both (2.3%, *n* = 3). Participants’ experience of combinations of IPV ranged from 11.7% to 19.5%, with 11.7% (*n* = 15) experiencing two types of IPV, 18% (*n* = 23) experiencing four types of IPV, and 19.5% (*n* = 25) experienced three types of IPV. However, 16.4% (*n* = 21) have experienced all types of IPV (Table 1).

Out of the 51 women who experienced physical violence, 23.5% (*n* = 12) had been injured as a result of a physical beating, 90% (*n* = 46) believed that beatings affected their physical or psychological health, and 62.7% (*n* = 32) believed that beatings affected their work as a result of loss of confidence in their capabilities or loss of their ability to concentrate. Half of physically abused women asked for help (*n* = 28). Most of them had spoken about physical abuse to their families (33.3%), followed by friends (13.7%), and only one participant had reported experiencing physical abuse to the police. Those who believed that their families had tried to help represented 29.4%. About a third of physically abused women (*n* = 15) did not ask for help for several reasons but mainly because of fear of losing custody of their children (*n* = 12).

As shown in Table 2, the participants had moderate psychological distress (M = 13.25, SD = 6.4) and perceived social support (M = 25.19, SD = 5.31). Specifically, the participants experienced moderate anxiety and depression (M = 5.48, SD = 2.6), social dysfunction (M = 6.39, SD = 3.10), and mild loss of confidence (M = 1.82, SD = 1.79). Participants reported greater perceived support from their husbands (M = 9.36, SD = 2.87) followed by their family (M = 8.23, SD = 2.50).

**Table 2:** Descriptive Statistics of Psychological Distress and Perceived Social Support and Their Subscales

Variables	M	SD	Range
**Psychological Distress**	13.25	6.40	0 – 36
Anxiety and depression	5.48	2.60	0 – 12
Social dysfunction	6.39	3.10	0 – 18
Loss of confidence	1.82	1.79	0 – 6
**Perceived Social Support**	25.19	5.31	12 – 36
Family	8.23	2.50	4 – 12
Friends	7.90	2.55	4 – 12
Husband	9.36	2.87	4 – 12

An independent samples t-test was used to determine whether psychological distress, anxiety and depression, social dysfunction, loss of confidence, and perceived social support differed between those who had experienced any type of IPV and those who had not (Table 3). The results indicated that those who had experienced physical abuse experienced significantly higher psychological distress (M = 15.05, SD = 6.82, *p* = 0.009), anxiety and depression (M = 6.29, SD = 2.57, *p* = 0.004), and loss of confidence (M = 2.57, SD = 1.90, *p* < 0.001).

**Table 3:** Mean Differences in Participants’ IPV victimization, Psychological Distress, and Perceived Social Support (*N* = 128)

		**Psychological distress**		**Anxiety & depression**		**Social dysfunction**		**Loss of Confidence**		**Perceived Social suport**	
		M (SD)	*p*	M (SD)	*p*	M (SD)	*p*	M (SD)	*p*	M (SD)	*p*
**IPV**	Yes No	13.67 (6.24) 9.16 (6.75)	**.010**	5.58 (2.58) 4.22 (2.63)	.066	6.49 (3.03) 5.41 (3.65)	.127	1.90 (1.79) .77 (1.39)	**.034**	25.10 (5.40) 26.09 (4.36)	.279
**Physical abuse**	Yes No	15.05 (6.82) 12.17 (6.10)	**.009**	6.29 (2.57) 4.98 (2.51)	**.004**	6.86 (3.38) 6.10 (2.99)	.100	2.57 (1.90) 1.47 (1.60)	** < .001**	24.06 (6.01) 26.23 (4.49)	**.016**
**Emotional abuse**	Yes No	14.87 (6.32) 11.51 (5.74)	**.002**	6.18 (2.57) 4.45 (2.36)	** < .001**	6.67 (3.19) 6.24 (2.86)	.224	2.31 (1.79) 1.17 (1.55)	** < .001**	24.55 (5.73) 26.12 (4.68)	.289
**Sexual abuse**	Yes No	14.98 (5.89) 11.46 (6.38)	** < .001**	6.22 (2.49) 4.55 (2.50)	** < .001**	6.66 (2.84) 6.23 (3.03)	.216	2.45 (1.78) 1.07 (1.51)	** < .001**	24.65 (5.17) 26.09 (5.10)	.073
**Control behavior**	Yes No	14.39 (7.19) 12.53 (5.44)	.050	5.77 (2.80) 5.25 (2.43)	.137	6.82 (3.31) 6.14 (2.82)	.107	2.36 (1.90) 1.42 (1.60)	**.002**	23.96 (5.61) 26.14 (4.93)	**.013**
**Financial abuse**	Yes No	14.07 (6.11) 11.68 (6.71)	**.022**	5.63 (2.54) 5.13 (2.73)	.161	6.60 (3.05) 5.97 (3.17)	.138	1.98 (1.82) 1.47 (1.68)	.073	24.79 (5.69) 25.90 (4.49)	.136
**Experience of IPV**	None One	9.16 (6.75) 10.37 (5.38)	**.002**	4.22 (2.63) 4.06 (2.34)	**.001**	5.41 (3.65) 5.71 (2.76)	.455	.77 (1.39) .90 (1.24)	** < .001**	26.09 (4.36) 25.51 (4.80)	**.024**
	Two	13.40 (5.86)		5.26 (2.25)		6.60 (2.72)		1.53 (2.03)		28.66 (4.01)	
	Three	14.84 (4.70)		6.32 (1.95)		6.56 (3.18)		1.96 (1.59)		25.37 (5.51)	
	Four	15.91 (6.08)		6.63 (2.53)		7.26 (3.00)		2.52 (1.60)		23.72 (5.13)	
	All	15.04(7.81)		6.00(3.02)		6.66(3.49)		3.11(1.99)		22.58(6.28)	

In addition, those who experienced either emotional or sexual abuse had higher psychological distress (*p* < 0.001), anxiety and depression (*p* < 0.001), and loss of confidence (*p* < 0.001). Women who experienced controlling behavior had a higher and more significant loss of confidence than their counterparts (*p* = 0.002). Participants who experienced financial abuse experienced psychological distress (*p* < 0.05). However, financial abuse did not result in significant differences in anxiety and depression, social dysfunction, or loss of confidence. In general, those experiencing IPV experienced higher psychological distress (M = 13.67, SD = 6.24, *p* = 0.01) and loss of confidence (M = 1.9, SD = 1.79, *p* = 0.03). As women experienced more different types of IPV, their psychological distress, anxiety, depression, and loss of confidence significantly increased (*p* = 0.002, 0.001, and < 0.001, respectively**)**. According to this study’s results, social dysfunction had no significant relationship with IPV (*p* > 0.05).

Regarding perceived social support, women who had experienced physical abuse and controlling behaviors perceived lower social support (*p* = 0.01) compared to their counterparts. However, IPV and emotional, sexual, and financial abuse were not significantly associated with perceived social support. Furthermore, the experience of IPV was negatively associated with perceived social support. As women experienced more different types of IPV, their perceived social support decreased significantly (*p* = 0.024).

Pearson’s correlation coefficient was used to determine the associations between anxiety and depression, social dysfunction, loss of confidence, support from family, support from friends, and support from husbands (Table 4). There was a positive and moderate correlation between anxiety and depression and social dysfunction (*r* = 0.503^**^, *p* < 0.01), loss of confidence (*r* = 0.412^**^), and support from family (*r* = 0.306, *p* < 0.01). Anxiety and depression were moderately and negatively correlated with support from husbands (>−0.456^**^). Participants with higher anxiety and depression scores had higher social dysfunction, lower self-confidence, and lower support from their husbands. Social dysfunction was positively and significantly correlated with loss of confidence (*r* = 0.395, *p* < 0.01) and support from family (*r* = 0.195, *p* < 0.05). The greater the social dysfunction participants reported, the greater their loss of confidence and support from family. Loss of confidence was significantly and negatively associated with support from husbands (*r* = −0.246, *p* < 0.05). The higher the support from husbands, the greater the participants’ confidence. Interestingly, anxiety and depression were not associated with support from friends (*p* > 0.05).

**Table 4:** The Association between Anxiety and Depression, Social Dysfunction, Loss of Confidence, Support of Family, Support of Friends, and Support of Husbands (*N* = 121)

Variables	1	2	3	4	5	6
Anxiety & depression	**1**					
Social dysfunction	.503^**^	**1**				
Loss of confidence	.412^**^	.395^**^	**1**			
Support of family	.306^**^	.195^*^	.082	**1**		
Support of friends	.077	-.093	-.043	.259^**^	**1**	
Support of husbands	-.456^**^	-.279^**^	-.246^*^	-.144	.018	**1**

Pearson’s correlation was used. ^*^p < .05; ^**^p < .01

Table 5 presents a comparison of different types of abuse (physical, emotional, sexual, controlling behavior, and financial) experienced by women based on various demographic factors. The results revealed that those women who do not work are more susceptible to both of emotional (*p* < 0.01) and financial abuse (*p* < 0.001) comparing to those who are employed. Husbands who work are more likely to engage in physical (*p* < 0.01) and emotional abuse (*p* < 0.001) compared to those who don't work. When it comes to arranged marriage, women who did not choose their husbands experience significantly more emotional abuse (*p* < 0.01) comparing to their counterparts. Also, wives and husbands with a history of child abuse or who have witnessed domestic violence increase the susceptibility to emotional and sexual forms of abuse.

**Table 5:** Comparison of Participants’ Sociodemographic Characteristics on Intimate Partner Violence Victimization

**Demographic Factor**		**IPV**		**Physical Abuse**		**Emotional Abuse**		**Sexual Abuse**		**Controlling** **Behavior**		**Financial Abuse**	
		M (SD)	*t*	M (SD)	*t*	M (SD)	*t*	M (SD)	*t*	M (SD)	*t*	M (SD)	*t*
**Working**	No	1.67 (.49)	1.47	1.45 (.50)	-.343	1.57 (.50)	2.22**	1.55 (.50)	1.57 *	1.44 (.50)	−0.63	1.73 (.45)	4.64***
	Yes	1.44 (.49)		1.48 (.51)		1.37 (.49)		1.41 (.49)		1.50 (.51)		1.33 (.47)	
**Husband** **Working**	No Yes	1.08 (.28) 1.07 (.25)	.16	1.02 (.12) 1.12 (.33)	−2.38**	1.04 (.20) 1.10 (.30)	−2.52***	1.07 (.26) 1.07 (.26)	-.017	1.08 (.28) 1.06 (.23)	0.61	1.05 (.21) 1.09 (.28)	−0.82
**Chose** **Husband**	No Yes	1.17 (.38) 1.42 (.49)	−2.10*	1.34 (.47) 1.49 (.50)	−1.61*	1.33 (.47) 1.43 (.49)	−1.18	1.36 (.48) 1.43 (.49)	−0.87	1.34 (.47) 1.46 (.50)	−1.44	1.36 (.49) 1.42 (.49)	−0.57
**Income is sufficient**	No	1.08 (.28)	**-** 3.73** *	1.37 (.48)	−1.060	2.93 (1.79)	−1.73*	3.02 (1.73)	−0.30	3.11 (1.67)	−1.27	3.21 (1.96)	−1.90*
	Yes	1.44 (.49)		1.47	.504	3.30 (1.61)		3.36 (1.74)		3.29 (1.80)		3.21 (1.62)	
**History of** **Child** **Abuse/Witnes sing domestic violence**	No Yes	1.75 (.45) 1.44 (.49)	−2.24*	1.42 (.49) 1.47	-.567 .504	1.55 (.50) 1.41 (.49)	−1.81*	1.54 (.50) 1.39 (.49)	−1.98*	1.46 (.50) 1.46 (.50)	−0.32	1.55 (.50) 1.43 (.49)	−1.44
**Husband's**	No	.30 (.48)	−1.19	.54 (.50)	.816	.33 (.47)	−2.74**	.36 (.48)	−2.30**	.44 (.50)	−0.84	.38 (.49)	−1.37
**History of Child Abuse Witnessing domestic violence**	Yes	.49 (.50)		.47 (.50)		.58 (.490)		.57 (.49)		. 52 (.50)		.52 (.51)	

^*^p < .05. **p < .01. ***p < .001

An independent samples t-test and one-way ANOVA were used to determine differences in psychological distress and perceived social support according to the participants’ demographic characteristics (Table 6). The results revealed that married women received higher social support (M = 25.7, SD = 4.75, *p* = 0.026) and had greater confidence (M = 1.67, SD = 1.73, *p* = 0.013) than divorced women. Additionally, those with higher education perceived greater social support (M = 26.3, SD = 5.98, *p* = 0.005) than those with high school or undergraduate degrees. Participants who believed that their income was adequate had lower psychological distress (M = 12.46, SD = 6.15, *p* = 0.04), anxiety and depression (M = 5.11, SD = 2.5, *p* = 0.03), and greater confidence (M = 1.50, SD = 1.68, *p* = 0.009) than those who believed that their monthly income was not adequate. Wives who lived with their partners in the same house perceived greater social support (M = 25.79, SD = 4.75, *p* = 0.009), lower psychological distress (M = 12.82, SD = 6.02, *p* = 0.04) and greater confidence (M = 1.55, SD = 1.59, *p* = 0.001) than those who do not live with their partners in the same house. Furthermore, those with a history of child abuse had greater anxiety and depression (M = 6.06, SD = 2.17, *p* = 0.02) than those without a history of child abuse and witnessing domestic violence. Regarding history of child abuse and witnessing domestic violence, there was no difference between the two groups in terms of perceived social support, psychological distress, social dysfunction, and loss of confidence.

**Table 6:** Comparison of Participants’ Sociodemographic Characteristics on Psychological Distress, and Perceived Social Support

Demographic Factors		Psychological distress		Anxiety and depression		Social dysfunction		Loss of confidence		Perceived social support	
		M (SD)	*t* or *f*	M (SD)	*t* or *f*	M (SD)	*t* or *f*	M (SD)	*t* or *f*	M (SD)	*t* or *f*
**Marital Status**	Married	13.15 (6.3)	.135	5.53 (2.48)	.676	6.43 (3.06)	.719	1.67 (1.73)	2.26**	25.73 (4.75)	2.54*
	Divorced	13.41 (8.1)		5.0 (3.41)		5.75 (3.76)		2.90 (1.81)		17.16 (8.18)	
**Education**	High school	13.43 (6.40)	.022	5.21 (2.96)	.549	6.80 (2.98)	.359	1.89 (1.77)	.276	22.39 (5.82)	5.51**
	Undergraduate degree	13.15 (6.68)		5.43 (2.66)		6.29 (3.19)		1.87 (1.81)		25.95 (4.57)	
	Higher education	13.33 (5.57)		6.00 (1.74)		6.14 (3.0)		1.55 (1.79)		26.36 (5.98)	
**Income is sufficient**	Yes No	12.46 (6.15) 14.40 (6.63)	**1.699** *	5.11 (2.50) 6.02 (2.68)	1.906*	6.23 (3.10) 6.61 (3.11)	.677	1.50 (1.68) 2.29 (1.85)	2.40**	25.59 (4.92) 24.58 (5.83)	1.02
**Living Together**	Yes No	12.82 (6.02) 15.55 (7.94)	1.76*	5.38 (2.48) 6.00 (3.17)	.949	6.30 (3.0) 6.85 (3.3)	.720			1.55 (1.59) 3.33 (2.11)	3.41**
**History of Child** **Abuse/** **Witnessing DV**	Yes No	25.84 (4.76) 24.81 (5.59)	1.020	13.93 (5.69) 12.83 (6.79)	.941	6.06 (2.17) 5.12 (2.78)	1.960*	6.39 (2.74) 6.38 (3.31)	.015	1.80 (1.70) 1.83 (1.85)	.085

*p<.05. ** *p*<.005. ***p<.001

## Discussion

In 2018, the WHO reported that one-third of women globally experienced various forms of violence at some point in their lives, with IPV being the most common [1]. Violence against women has received widespread recognition as a grievous violation of human rights, and as a public and clinical concern. This does not significantly increase the body of evidence highlighting the prevalence of IPV among women in Saudi Arabia. Despite its prevalence, IPV against women in Saudi Arabia remains a contentious topic and cultural taboo. This study evaluated the prevalence of IPV victimization among women in Saudi Arabia and its association with psychological health outcomes. We also investigated the effects of social support on experiencing IPV and women’s psychological health.

The results of our study revealed that IPV was prevalent among participants, which includes emotional (57.8%), sexual (53.9%), controlling behavior (43.8%), and physical abuse (39.8%), and a minimum of 25% of women had experienced at least one form of IPV. Notably, nearly 20% of the participating women experienced a minimum of three forms of abuse. According to a previous study in Saudi Arabia [28], abuse affects at least one-third of women, underscoring the need for strict policies and regulations to combat violence and support victims [29]. In our study, women reported financial abuse as the most common type of abuse, accounting for 65.6%. A study by [30] indicated that the prevalence of economic violence in Saudi Arabia against women was 26%. These findings highlight the importance of enhancing the financial independence of women. The prevalence of physical violence was lower than that in a past study conducted in Saudi Arabia, whereas the prevalence of emotional and sexual abuse was higher [28].

Considering the detrimental effects of physical violence on women’s health and well-being, approximately 35% of the participants refrained from disclosing violent incidents to anyone. Apprehensions regarding social isolation and social stigma, perceived as threats to family stability, may rationalize this behavior [29]. This study found that women who had experienced IPV presented with psychological distress, such as anxiety, depression, and lack of confidence. This result is consistent with previous studies showing that women who experience IPV are more likely to experience psychological distress [31, 32].

In a meta-analysis conducted by [33] among 250,599 women across 46 countries, it was found that exposure to IPV significantly increased the risk of adverse outcomes such as depression, anxiety, and psychological distress. This finding highlights the need for governmental and non-governmental organizations to support the role of social and rehabilitation programs and services in supporting victims of violence. Many societies may accept and justify controlling behavior; however, reports suggest that its negative psychological effects could surpass those of physical violence [34]. Our results showed that women who suffered from partner controlling behaviors reported a lack of confidence.

Our findings indicated that certain socio-demographic factors can either increase or decrease the risk of IPV. Working women were less likely to experience IPV, specifically physical, sexual, and financial abuse, suggesting that economic independence may contribute to lower vulnerability to these forms of abuse. This finding contradicts a study conducted in Riyadh in 2018, which revealed that abuse was higher among employed women [28]. This variation may be attributed to the increasing number of employed women in the labor market in recent years unlike previously [8]. In contrast, employed husbands are more likely to physically and emotionally abuse their wives, possibly due to stress, power dynamics, or other factors associated with employment. Being victims of childhood abuse, on both the wife's and husband's sides, can increase the risk of emotional and sexual abuse. Our findings align with those of the Centers for Disease Control and Prevention, which highlighted that unemployment, financial stress, a history of child abuse, and witnessing domestic violence can increase the likelihood of experiencing IPV [35].

Surprisingly, despite the high prevalence of IPV in this study, women reported substantial perceived support from their husbands, followed by their friends and then family. This reflects social and cultural norms that regard violence as a form of discipline and an inward dilemma that ought to be confined to the family unit [29]. This might also be due to the perception of the husband as the leader of the household and a source of income.

However, women who experienced physical abuse and controlling behaviors perceived lower social support. Social support has been shown to be a significant factor in buffering the impact of IPV [29, 36]. This has increased the urgency of providing abused women with social services that are accessible, effective, and reliable [30]. Specifically, our results demonstrated that low perceived support from husbands correlated with psychological distress, which might induce feelings of neglect and insecurity. However, interestingly, our findings showed that family support did not buffer against IPV or its impact. In contrast, family support increased anxiety and depression, and social dysfunction. This could be due to families’ objection to divorce due to the stigma associated with it and their pushing women to tolerate abuse, as it is normalized in society. This is supported by the findings, as only 39% of abused women disclosed physical abuse to a family member, while 16% did not, perhaps because they were afraid of having a negative effect on their family’s reputation.

Married women reported higher levels of perceived social support, particularly when they lived with their spouse. People commonly recognize marriage as a factor that positively affects social support, stability, and economic security [37]. However, women who experienced living away from their husbands were more prone to reporting psychological distress and diminished self-confidence. Separation may lead to feelings of isolation resulting in anxiety, depression, and low self-esteem [38].

Regarding educational level, the postgraduate group had the highest perceived social support. One possible explanation is that having a high educational level may increase access to social support by fostering a greater capacity for reciprocal assistance, thereby potentially improving the likelihood of receiving support. Financial strain raises concerns regarding family well-being [39]. Our findings indicate that perceived income inadequacy increases psychological distress, anxiety, depression, and loss of confidence. Literature supports the relationship between economic stress and health status, as financial hardship leads to psychological problems and affects mental health [39–41].

Based on the results obtained, women with a history of child abuse and witnessing domestic violence had higher levels of anxiety and depression than those with other encounters. A recent systematic review examined the effects of child abuse on women’s health. The results confirmed that women with a history of child abuse were more likely to suffer from depression, anxiety, physical and mental dysfunction, and early mortality [42]. Furthermore, a number of researchers highlighted the relationship between childhood experiences and IPV victimization, arguing that a history of child maltreatment distorts perceptions and beliefs about oneself and others and increases maladaptive coping strategies, which in turn influence interactions with intimate partners [42].

### Study strengths and limitations

Although this is a novel study, as it captured data from a wide range of participants living in Saudi Arabia, it still has several limitations. The data were collected from different regions in SA; however, most of the participants were from major cities in Saudi Arabia, thus limiting the generalizability of the findings to rural areas. The cross-sectional design and snowball sampling method prevented the authors from determining causality. Using media platforms to recruit participants may have contributed to the higher prevalence of intimate partner violence (IPV) in our study compared to previous studies conducted in Saudi Arabia. Abused women might be more inclined to fill out the online survey. Furthermore, as the questionnaire included several sensitive questions, the participants may have responded to these items in a socially desirable manner. However, to avoid such pitfalls, the participants were informed that identifying information would not be collected and that their responses would remain anonymous.

### Implications and recommendations

This study has several important implications for practice, future research, and policy. Our findings are consistent with those of previous Saudi-based studies and indicate that IPV is prevalent and requires substantial action plans from legislators, governmental bodies, social services, and protection services. Enhancing and broadening the scope of services available to women with IPV is crucial, including raising awareness of violence against women, which could aid the movement towards women's rights to respect, dignity, protection, and empowerment. Furthermore, advocacy and screening programs are recommended to make them easily accessible and ensure that they reach all women. We recommend conducting a national-level training program for all newlywed couples to acquire conflict-resolution skills, which may prevent the escalation of violence. Further research should pinpoint additional variables that could potentially contribute to IPV, including husbands’ traits and experiences. The physical, mental, and psychological health consequences of IPV require further investigation. Conducting empirical studies on IPV from the male perspective is another valuable avenue for future research. Our findings can also benefit countries with similar societal and cultural norms, such as Middle Eastern and Gulf countries. Finally, this study is valuable for developing evidence-based policies to address the impact of IPV on women's psychological health and emphasizes the importance of perceiving continuous support.

## Conclusions

This is one of the few studies evaluating the prevalence of IPV among women in Saudi Arabia and its association with psychological health outcomes and social support. The findings of this study unequivocally demonstrate that IPV is a prevalent and significant social and health problem requiring attention and investigation. IPV can cause psychological distress, anxiety, depression, and loss of confidence and can be buffered by social support. Our findings highlight the urgency of adopting strict policies and interventions to prevent violence. Furthermore, evidence-based interventions must be developed and evaluated to support victims. Based on the study’s findings, more attention should be paid to divorced and separated women, women who struggle financially, and women with a history of childhood abuse.

## Data Availability

The data supporting the findings of this study are available from the corresponding author (Adnan Innab) upon reasonable request.

## Abbreviations

IPV: Intimate partner violence
STROBE: Strengthening the Reporting of Observational Studies in Epidemiology
GHQ: General Health Questionnaire
MSPSS: Multi-Dimensional Scale of Perceived Social Support
WHO: World Health Organization
ANOVA: Analysis of Variance
